# Joint detection of multiple immunohistochemical indices and clinical significance in breast cancer

**DOI:** 10.3892/mco.2013.111

**Published:** 2013-05-08

**Authors:** EN-QI QIAO, MINGHUA JI, JIANZHONG WU, JIAN LI, XINYU XU, RONG MA, XIAOHUA ZHANG, YUEJUN HE, QUANBIN ZHA, XUE SONG, LIWEI ZHU, JI-HAI TANG

**Affiliations:** 1 Departments of General Surgery, Affiliated to Nanjing Medical University, Nanjing, Jiangsu 210009;; 2 Radiotherapy, Affiliated to Nanjing Medical University, Nanjing, Jiangsu 210009;; 3 Research Center for Clinical Oncology, Affiliated to Nanjing Medical University, Nanjing, Jiangsu 210009;; 4 Department of Pathology, Jiangsu Cancer Hospital, Affiliated to Nanjing Medical University, Nanjing, Jiangsu 210009;; 5 Nanjing Medical University, Nanjing, Jiangsu 210029;; 6 Xuzhou Medical College, Xuzhou, Jiangsu 221000, P.R. China

**Keywords:** breast carcinoma, clinicopathological characteristics, immunohistochemical index, breast cancer subtype

## Abstract

Breast cancer is one of the most common malignancies in women. This study was conducted to analyze the association between the expressions of eight immunohistochemical (IHC) indices and clinicopathological characteristics in breast cancers (BCs) and investigate the clinical significance. IHC Envision ldpe-g-nvp was used to detect the expression of estrogen receptor (ER), progesterone receptor (PR), human epidermal growth factor receptor-2 (HER2), vascular endothelial growth factor (VEGF), epidermal growth factor receptor (EGFR), p53, type II topoisomerase (TOPO II) and Ki-67 in postoperative paraffin blocks of 286 cases of invasive BC and statistically analyzed their correlations with clinicopathological characteristics. The positive rates of ER, PR, HER2, VEGF, p53, EGFR, TOPO II and Ki-67 expression were 62.24, 41.96, 57.34, 53.85, 81.82, 46.85, 54.55 and 69.93%, respectively. ER expression was negatively correlated with age, tumor size and histological grade (P<0.05) and PR expression was negatively correlated with age and histological grade (P<0.05). Among the ER, PR and c-erbB-2 statuses, a significant correlation was observed between ER expression and PR status (P=0.0000), whereas the expression of ER and PR exhibited a negative correlation with HER2 status (P<0.05). We also demonstrated a significant correlation between EGFR expression and lymph node metastasis (P=0.0240), p53 expression and tumor size (P=0.0300), p53 and Ki-67 expression and histological grade (P<0.05) and the expressions of VEGF, EGFR, p53, TOPO II, Ki-67 and HER2 status (P<0.05). In addition, the Luminal B and HER2/neu subtypes exhibited a close correlation with age (P<0.01), while the HER2/neu and triple-negative subtypes were positively correlated with poor histological grade (P<0.05). In conclusion, there is a definite correlation between IHC indices and clinicopathological characteristics in BCs. Combined detection of these indices may be significant in the evaluation of biological behavior and prognosis of BC and thus in the diagnosis and comprehensive treatment of this disease.

## Introduction

Breast cancer (BC) is one of the most common malignancies in women. There are >1,000,000 new cases diagnosed annually worldwide. A marked increase in incidence occurs annually and in particular among women of younger age, which poses a serious threat to women’s physical and mental health (1). The occurrence, progression and prognosis of various types of cancer are complex processes, involving numerous factors, genes and proteins. Therefore, it is difficult to determine the biological behavior and prognosis of BC based on the assessment of a single factor.

Immunohistochemical (IHC) detection has become essential to many malignancies and plays a key role in tumor diagnosis, treatment and prognostic assessment. In this study, we collected 286 cases of invasive BC, confirmed at our pathology laboratory, detected the expression of estrogen receptor (ER), progesterone receptor (PR), human epidermal growth factor receptor-2 (HER2), vascular endothelial growth factor (VEGF), epidermal growth factor receptor (EGFR), p53, type II toposomerase (TOPO II) and Ki-67 proteins by IHC and analyzed the associations between these indicators and the clinicopathological characteristics, designed to further investigate the associations of the expressions of hormone receptors, oncogenes and proteins with BC biological behavior and prognosis, in order to guide clinical diagnosis and comprehensive treatment.

## Materials and methods

### Materials

Tissue samples from 286 patients with invasive BC were collected in Jiangsu Cancer Hospital, Affiliated to Nanjing Medical University, between February, 2011 and July, 2012. All the samples were primary cancers, with complete medical records and a confirmed diagnosis. The patients had not received any treatment prior to surgery. All patients were female, aged 28–79 years (median, 51 years). A total of 146 cases were >50 years of age and the remaining 140 cases were ≤50 years of age. The tumor size was >2 cm in 122 cases and ≤2 cm in 164 cases. Lymph node metastasis was identified in 134 cases and 152 cases presented with non-metastatic lymph nodes, as determined by IHC. Histological grades were classified according to the WHO histological classification standards for evaluation of BC (2003). Among the cases there were 24 grade I, 184 grade II and 78 grade III cases. Monoclonal antibodies against ER, PR, HER2, VEGF, EGFR, p53, TOPO II and Ki-67, as well as IHC kits, were purchased from Zhongshan Jinqiao Biotechnology Co., Ltd. (Beijing, China).

### Methods

**Figure 1:**
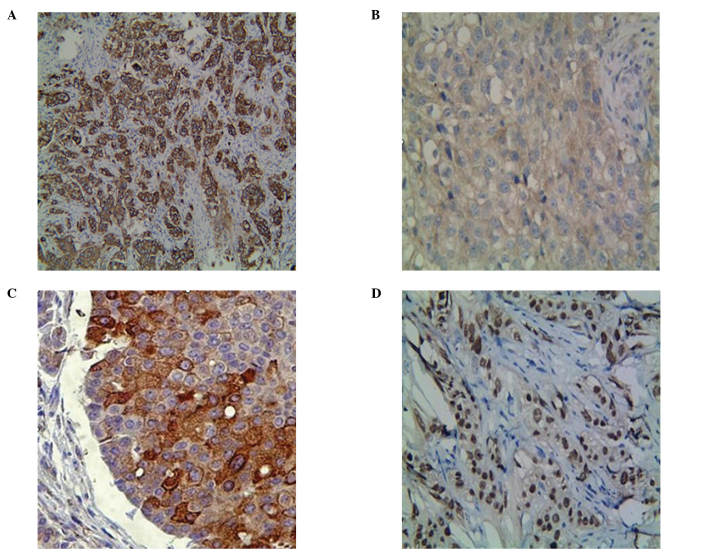
Positive expressions of immunohistochemical (IHC) indices in invasive breast cancer tissues. Indices that (A) exist only in the membrane (human epidermal growth factor receptor-2, HER2), (B) are present mainly in the cytoplasm (epidermal growth factor receptor, EGFR), (C) are present in both the cytoplasm and the membrane (vascular endothelial growth factor, VEGF), (D) are detected only in the nucleus [estrogen receptor (ER), progesterone receptor (PR), p53, type II topoisomerase (TOPO II) and Ki-67] are shown. Images were IHC 3+ or fluorescence *in situ* hybridisation (FISH)-positive. EnVision (magnification, ×100/×400).

The samples were fixed in 10% neutral formalin, desiccated and embedded in paraffin, then sliced into 4-*μ*m sections. Envision ldpe-g-nvp was used as the staining method. The primary antibody dilution and process of staining were performed according to the manufacturer’s instructions. Equivalent phosphate-buffered saline (PBS) was used as a negative control for primary antibodies. The results were observed under a microscope. The staining of HER2 was mainly localized in the membrane, EGFR was localized mainly in the cytoplasm and to a limited extent in the membrane, VEGF was localized in the cytoplasm and the membrane, whereas ER, PR, p53, TOPO II and Ki-67 were localized in the nucleus (Fig. 1). Cells were classified according to the positive rate and colour intensity as follows: negative (−), number of positive cells <5%; weak positive (+), pale brown particles, number of positive cells 6–25%; positive (++), brown particles, number of positive cells 26–50%; strong positive (+++), dark brown particles, number of positive cells >50%. The results of the assessment of HER2-positive cells were in accordance with those of a previous study (2).

### Statistical analysis

Statistical analysis was performed with SPSS software v. 16.0 and the enumeration data were compared with the Chi-square (χ^2^) test. P≤0.05 was considered to indicate a statistically significant difference.

## Results

### Association between the expressions of ER, PR and HER2 in breast cancers (BCs) and clinicopathological characteristics

As shown in Table I, the positive rates of ER or PR, as well as the double-positive rate of ER and PR, were highest in the lower age group (69.51, 55.71 and 51.43%, respectively). ER expression exhibited a negative correlation with age, tumor size and histological grade (χ^2^=4.9284, P=0.0260; χ^2^= 4.3281, P=0.0370; and χ^2^=4.1706, P=0.0410, respectively), PR expression exhibited a negative correlation with age and histological grade (χ^2^=10.6550, P=0.0011 and χ^2^=4.1649, P=0.0410, respectively) and the double-positive expression of ER and PR also exhibited a negative correlation with age and histological grade (χ^2^=8.6617, P=0.0033 and χ^2^=4.1141, P=0.0430, respectively). However, there was no statistically significant difference among the indices in lymph node metastasis. The positive rate of HER2 was highest in the lymph node metastasis group (62.69%); although no correlation with age, tumor size, lymph node metastasis and histological grade was evident.

### Association of the expressions of ER, PR and HER2 in BCs

As shown in Table II, the positive rates of ER and PR in the HER2-negative cases (80.33 and 54.10%, respectively) were significantly higher compared to the HER2-positive cases (48.78 and 32.93%, respectively). The expression of PR was closely associated with the ER status (χ^2^=42.5299, P=0.0000) and the expressions of ER and PR were negatively correlated with HER2 (χ^2^=14.8123, P=0.0001 and χ^2^= 6.4381, P= 0.0110, respectively).

### Association between the expression of VEGF, EGFR, p53, TOPO II and Ki-67 in BCs and clinicopathological characteristics

As shown in Table III, the expression of EGFR was highest in the lymph node metastasis group (89.55%) and exhibited a positive correlation with lymph node metastasis (χ^2^=5.0690, P=0.0240). The positive rates of p53 and Ki-67 were highest in the poor histological grade group (61.54 and 84.62%, respectively), p53 expression was correlated with tumor size and histological grade (χ^2^=4.7314, P=0.0300 and χ^2^=4.6443, P=0.0310, respectively) and Ki-67 expression was correlated with histological grade (χ^2^=5.5000, P=0.0190). The expressions of VEGF and TOPO II did not statistically differ with age, tumor, lymph node metastasis and histological grade.

### Association between the expressions of ER, PR and HER2 and the expressions of VEGF, EGFR, p53, TOPO II and Ki-67 in BCs

As shown in Table IV, the expression of VEGF exhibited a negative correlation with ER and PR status (χ^2^=5.7384, P=0.0166 and χ^2^=10.0099, P=0.0016, respectively) and a positive correlation with HER2 expression (χ^2^=5.3917, P=0.0202). The expressions of EGFR, p53, TOPO II and Ki-67 were closely associated with HER2 status (χ^2^=9.1730, P=0.0020; χ^2^=18.1705, P=0.0000; χ^2^=14.6526, P=0.0000 and χ^2^=7.9717, P=0.0050, respectively).

### Association among the expressions of VEGF, EGFR, p53, TOPO II and Ki-67 in BCs

As shown in Table V, the expression of TOPO II exhibited a negative correlation with EGFR and a positive correlation with p53 and Ki-67 (χ^2^=4.40160, P=0.03600; χ^2^=10.12500, P=0.00150 and χ^2^=14.66030, P=0.00013, respectively). The expression of Ki-67 was significantly correlated with p53 (χ^2^=11.17360, P=0.00083). The association between the expression of VEGF and the levels of EGFR, p53, TOPO II and Ki-67 was not statistically significant.

### Association between the IHC approximate molecular subtypes of BC and clinicopathological characteristics

As shown in Table VI, in all the cases, the percentages of Luminal A, Luminal B, HER2/neu and triple-negative subtype were 20.98, 45.45, 26.57 and 9.09%, respectively. The proportion of Luminal A subtype was highest in the non-metastatic lymph node group (25%) and decreased with increasing histological grade; however, there was no statistically significant difference in these data. The proportion of Luminal B subtype was highest in the lower age group (58.57%), with a statistically significant difference (χ^2^=9.5156, P=0.0020). However, it was higher in the poor histological grade group, although the difference was not statistically significant. The proportion of the HER2/neu subtype was highest in the poor histological grade group (38.46%) and exhibited a statistically significant difference in age and histological grade (χ^2^=8.2871, P= 0.0040 and χ^2^=3.8841, P=0.0490, respectively). The proportion of the triple-negative subtype was highest in the poor histological grade group (17.95%), the difference was statistically significant (χ^2^=5.0910, P=0.0240) and exhibited no correlation with age, tumor size or lymph node metastasis.

## Discussion

Several tumors are hormone-dependent and BC is a typical example. ER and PR play important roles in the growth and differentiation of BCs while their expression levels are decisive factors guiding the endocrine treatment of BCs, and important prognostic markers. Our data demonstrated that the positive rates of ER and PR were 62.24 and 41.96%, which was in agreement with the findings of a previous study (3). Furthermore, this study demonstrated that ER expression decreased with increasing histological grade, indicating that the lower the tumor cell differentiation, the lower the estrogen dependence, thus affecting the sensitivity to hormone therapy. The association between ER expression and lymph node metastasis was diverse in previous studies (4,5). Our results demonstrated that the expression of ER was negatively correlated with age and tumor size and had no correlation with lymph node metastasis. ER expression may not be an independent prognostic factor, as PR and ER are steroid hormone receptors that belong to the nuclear receptor superfamily and PR is a derivative of estrogen and ER combination. A previous study showed that the tumor-free survival of PR-positive BC patients was significantly longer compared to those of PR-negative BC patients (6). PR may downregulate the expression of breast cancer resistance protein (BCRP) and increase chemosensitivity (7). Our study showed that the expression of PR exhibited statistically significant differences with age and histological grade, which may be related to the presence of independent regulatory pathways affected by PR but not ER. Data also showed that PR expression was decreased in lymph node metastasis. Further analysis demonstrated that the double-positive rate of ER and PR was 39.16% and it was significantly different in lymph node metastasis and histological grade. Thus, it is essential that ER and PR are assessed as a whole to determine patient prognosis. With the development of the tumor, part of ER-positive and/or PR-positive patients start exhibiting hormone therapy resistance and this ‘escape’ mechanism of ER and PR requires further investigation (8,9).

HER2, a proto-oncogene, also known as c-erbB-2 or HER2/neu, located on chromosome 17q21, is considered to be closely associated with the occurrence and development of BC (10). Under normal physiological conditions HER2 is inactive; however, once activated, it may enhance tumor invasion and metastasis and increase the degree of malignancy (11). A previous study reported that HER2 overexpression in BCs indicated poor prognosis (12). Schillaci *et al* (13) identified NuclErbB-2 positivity as a significant independent predictor of worse overall survival (OS) in patients with MembErbB-2 overexpression. Our results demonstrated that the expressions of ER and PR were significantly correlated with c-erbB-2 status, which was in agreement with findings reported by previous studies (14,15). The presence of HER2 reduced the efficacy of endocrine therapy, therefore, the efficacy of endocrine therapy was increased with targeted inhibition of the expression of HER2 (16). Our study identified an association between HER2 status and the expressions of ER and PR: HER2 overexpression may exert an inhibitory effect on the expressions of ER and/or PR. Our results also demonstrated that the expression of HER2 was enhanced with increasing age and lymph node metastasis; although, these data were not statistically significant. The anti-HER2 monoclonal antibodies have been used in the clinical treatment of BC patients. However, due to the variations of chromosome 17 and HER2/neu genetic heterogeneity, IHC and FISH assay assessment of HER2 expression is required prior to targeted therapy (17).

Thus far, VEGF is known as the most important angiogenesis-promoting factor, is highly expressed in several malignant tumors and plays an important role in the occurrence, development and metastasis of tumors (18,19). Combining VEGF with p53 status may result in a better prognostic prediction in BC patients (20). Our results demonstrated that the positive rate of VEGF expression in BCs was 53.85% and the positive expression levels were higher in the lymph node metastasis group compared to the non-metastatic group; however, there were no statistically significant differences with age, tumor size, lymph node metastasis and histological grade, a finding consistent with those of Jobim *et al* (21). Our data also showed that VEGF expression was negatively correlated with ER and PR status and positively correlated with HER2 status, which was in agreement with the findings of Linderholm *et al* (22), who reported that triple-negative BCs (TNBCs) have a higher VEGF level compared to non-TNBC.

EGFR belongs to the tyrosine kinase receptor family and is associated with cell growth, proliferation and differentiation. It was previously demonstrated that 50–70% of TNBCs express EGFR (23) and EGFR overexpression was associated with TNBCs and unfavorable prognosis (24). TNBCs with a low EGFR expression exhibited a lower incidence of metastasis (25). Our data showed that the positive rate of EGFR expression in BCs was as high as 81.82% and its expression was significantly correlated with lymph node metastasis and HER2 status (P<0.05). Its expression in ER- or PR-positive groups was lower compared to that in ER- or PR-negative groups; however, the difference was not statistically significant, which is consistent with the findings of a previous study (26).

The p53 gene is located on human chromosome 17p13.1 and its main function is to induce cell cycle arrest and apoptosis and to promote cell differentiation. The p53 gene may be found as wild-type or mutant and the mutant form prevents the wild-type p53 gene from inhibiting tumor formation, leading to cell transformation and cancerization (27). The five-year survival rate of patients with p53-positive BC was significantly lower compared to that of p53-negative BC patients (28). Our results showed the positive rate of p53 protein is 46.8% in BCs, which was statistically correlated with tumor size and histological grade and its expression in the lymph node metastasis group was higher compared to that in the non-metastatic group, although the difference was not statistically significant, which was in agreement with the above viewpoints and the findings of a previous study (29). Futhermore, we observed that the p53 positive expression rates in the ER- or PR-positive groups were lower compared to those in the ER- or PR-negative groups and had a positive correlation with HER2 status, which was in agreement with the findings of previous studies (29,30), indicating that they may exert a synergistic effect on endocrine and targeted therapies.

The TOPO II gene is located on human chromosome 17q21.3 and plays a key role in DNA melting, linking, repair and replication. Our study demonstrated that TOPO II expression in BC tissues was higher in the poor histological grade and lymph node metastasis groups, a finding consistent with those reported by a previous study (31). Therefore, the level of TOPO II expression may reflect the proliferation and metastatic status of BCs, guiding clinical treatment. Our data demonstrated that TOPO II expression exhibited a significant positive correlation with HER2 status, which was in agreement with previous findings (32). We also observed that TOPO II expression was significantly correlated with the status of EGFR, p53 and Ki-67 (P<0.05), indicating the presence of interactions among these indicators, regulating the progression of tumorigenesis and metastasis of BCs.

Ki-67 is a nuclear antigen related to cell proliferation, only expressed by the proliferating cell nucleus. The expression of Ki-67 is an IHC index detecting cell proliferative activity (33). The levels of Ki-67 expression in BCs were significantly higher compared to those in benign breast lesions and were elevated with increasing cancer cell atypia, which was significant for evaluating the prognosis of BCs (34,35). Our data showed that Ki-67 expression was significantly correlated with poor histological grade and was higher in the lymph node metastasis group compared to the non-metastatic group, indicating that Ki-67 expression is associated with tumor cell differentiation, invasion and metastasis. Ki-67 expression exhibited a significantly positive correlation with HER2 status, more pronounced in the ER- or PR-negative BCs compared to the ER- or PR-positive BCs, which was in agreement with previous findings (36). Furthermore, our study also demonstrated that the expression of Ki-67 exhibited a significantly positive correlation with cell proliferation-associated nuclear proteins, p53 and TOPO II, suggesting that Ki-67 is closely related to the proliferation of BC cells.

To divide BCs into various subtypes is an inevitable trend for the investigation and treatment of BCs. In accordance with the intrinsic genotyping, BC is divided into four subtypes: Luminal A, Luminal B, HER2/neu and basal-like subtype. Luminal B type was further subdivided into the high Ki-67 expression and HER2-positive subtypes according to Cheang *et al* (37). However, most experts agree that practically, the results of the detection of ER, PR, HER-2 and Ki-67 indices, equally divide BCs into four types as close substitutes, including Luminal A, Luminal B, HER2/neu and triple-negative subtypes (Table VII). The triple-negative subtype exhibits an almost 80% overlap with the basal-like subtype. Xue *et al* (38) collected a total of 5,809 patients with invasive ductal carcinoma and retrospectively analyzed their clinicopathological characteristics and survival rates. Of these patients, 31.1% were Luminal A, 30.4% were Luminal B (high Ki-67), 13.1% were Luminal B (HER2/neu+), 9.0% were HER2/neu and 16.5% were triple-negative subtype. The patients with Luminal B subtype were mainly distributed in the lower age group (<43 years old), the HER2/neu subtype was closely associated with tumor size, lymph node-positive status and vascular invasion and the triple-negative BCs were associated with poor histological grade (38). Our study results were mostly consistent with these findings, however, there were also certain differences: for example, the ratio of the patients with the HER2/neu subtype was 26.57% and it exhibited a significantly negative correlation with age, a finding that was in agreement with those of Munjal *et al* (39).

In conclusion, the development of malignancy is a process involving multiple factors, genes and steps. Our study demonstrated that ER, PR, HER2, VEGF, EGFR, p53, TOPO II and Ki-67 exhibited high expression levels in invasive BCs and they also exhibited certain interactions. As regards the associations between these indices and the clinicopathological characteristics of BCs, our results were mostly consistent with those of related previous studies, with certain differences and novel observations. Joint detection of various IHC indices may more accurately determine the biological characteristics and predict the prognosis of BCs, as well as provide a theoretical basis for the diagnosis and treatment of BCs (40). The ongoing scientific investigation may lead to the identification of novel IHC indices associated with BCs.

## Figures and Tables

**Table I.** Association between the expressions of ER, PR and HER2 in BCs and clinicopathological characteristics [cases, (%)].

Clinicopathological characteristics	No.	ER		PR		HER2		ER and PR	
		(+)	P-value	(+)	P-value	(+)	P-value	(+)	P-value
Age (years)									
≤50	140	100 (71.42)	0.0260^a^	78 (55.71)	0.0011^b^	74 (52.86)	0.2280	72 (51.43)	0.0033^b^
>50	146	78 (54.80)	4.9284	42 (28.76)	10.6550	90 (61.64)	-	40 (20.40)	8.6617
Tumor size (cm)									
≤2	164	114 (69.51)	0.0370^a^	74 (45.12)	0.3740	92 (56.10)	0.7270	70 (42.68)	0.3170
>2	122	64 (52.45)	4.3281	46 (37.70)	-	72 (59.02)	-	42 (34.42)	-
Lymph node metastasis									
Positive	134	82 (61.19)	0.8090	50 (37.31)	0.2900	84 (62.69)	0.2250	44 (32.84)	0.1460
Negative	152	96 (63.16)	-	70 (46.05)	-	80 (52.63)	-	68 (44.74)	-
Histological grade									
I–II	208	140 (67.31)	0.0410^a^	98 (47.12)	0.0410^a^	118 (56.73)	0.8090	92 (44.23)	0.0430^a^
III	78	38 (48.71)	4.1706	22 (28.21)	4.1649	46 (58.97)	-	20 (25.64)	4.1141

a P≤0.05,

b P≤0.01, the χ^2^ values appeared when P≤0.05. BCs, breast cancers; ER, estrogen receptor; PR, progesterone receptor; HER2, human epidermal growth factor receptor-2.

**Table II.** Association among the expressions of ER, PR and HER2 in BCs [cases, (%)].

Indices	No.	PR		HER2	
		(+)	P-value	(+)	P-value
ER					
(+)	178	112 (66.29)	0.0000^b^	80 (44.94)	0.0001^b^
(−)	108	8 (7.41)	42.5299	84 (77.78)	14.8123
PR					
(+)	120	-	-	54 (45.00)	0.0110^a^
(−)	166	-	-	110 (66.27)	6.4381
HER2					
(+)	164	54 (32.93)	0.0110^a^	-	-
(−)	122	66 (54.10)	6.4381	-	-

a P≤0.05,

b P≤0.01, the χ^2^ values appeared when P≤0.05. BCs, breast cancers; ER, estrogen receptor; PR, progesterone receptor; HER2, human epidermal growth factor-2.

**Table III.** Association between the expressions of VEGF, EGFR, p53, TOPO II and Ki67 in BCs and clinicopathological characteristics [cases, (%)].

Clinicopathological characteristics	No.	VEGF		EGFR		p53		TOPO II		Ki-67	
		(+)	P-value	(+)	P-value	(+)	P-value	(+)	P-value	(+)	P-value
Age (years)											
≤50	140	68 (48.57)	0.2150	112 (80.00)	0.3047	64 (45.71)	0.7893	70 (50.00)	0.2850	104 (74.29)	0.2660
>50	146	86 (58.90)	-	122 (83.56)	-	70 (47.95)	-	86 (58.90)	-	96 (65.75)	-
Tumor size (cm)											
≤2	164	86 (52.44)	0.6960	132 (80.49)	0.6320	64 (39.02)	0.0300^a^	86 (52.44)	0.5580	110 (67.07)	0.3880
>2	122	68 (55.74)	-	102 (83.61)	-	70 (57.38)	4.7314	70 (57.38)	-	90 (73.77)	-
Lymph node metastasis											
Positive	134	80 (59.70)	0.1870	120 (89.55)	0.0240^a^	56 (41.79)	0.2550	70 (52.24)	0.6030	96 (71.64)	0.6750
Negative	152	74 (48.68)	-	114 (75.00)	5.0690	78 (51.32)	-	86 (56.58)	-	104 (68.42)	-
Histological grade											
I-II	208	110 (52.88)	0.7060	168 (80.77)	0.5950	86 (41.35)	0.0310^a^	108 (51.92)	0.3040	134 (64.42)	0.0190^a^
III	78	44 (56.41)	-	66 (84.62)	-	48 (61.54)	4.6443	48 (61.54)	-	66 (84.62)	5.5000

a P≤0.05,

b P≤0.01, the χ^2^ values appeared when P≤0.05. BCs, breast cancers; VEGF, vascular endothelial growth factor; EGFR, epidermal growth factor receptor; TOPO II, type II topoisomerase.

**Table IV.** Association between the expressions of ER, PR and HER2 and the expressions of VEGF, EGFR, p53, TOPO II and Ki67 in BCs [cases, (%)].

Indices	No.	VEGF		EGFR		p53		TOPO II		Ki-67	
		(+)	P-value	(+)	P-value	(+)	P-value	(+)	P-value	(+)	P-value
ER											
(+)	178	82 (46.07)	0.0166^a^	144 (80.90)	0.7140	82 (46.07)	0.8090	98 (55.06)	0.8750	120 (67.42)	0.4000
(−)	108	72 (66.67)	5.7384	90 (83.33)	-	52 (48.15)	-	58 (53.70)	-	80 (74.07)	-
PR											
(+)	120	46 (38.33)	0.0016^b^	92 (76.67)	0.1740	52 (43.33)	0.4730	62 (51.67)	0.5040	78 (65.00)	0.2740
(−)	166	108 (65.06)	10.0099	142 (85.54)	-	82 (49.40)	-	94 (56.63)	-	122 (73.49)	-
HER2											
(+)	164	102 (62.20)	0.0202^a^	148 (90.24)	0.0020^b^	102 (62.20)	0.0000^b^	112 (68.29)	0.0000^b^	130 (79.27)	0.0050^b^
(−)	122	52 (42.62)	5.3917	86 (70.49)	9.1730	32 (26.23)	18.1705	44 (36.07)	14.6526	70 (57.38)	7.9717

a P≤0.05,

b P≤0.01, the χ^2^ values appeared when P≤0.05. BCs, breast cancers, ER, estrogen receptor; PR, progesterone receptor VEGF, vascular endothelial growth factor; EGFR, epidermal growth factor receptor; TOPO II, type II topoisomerase, HER2; human epidermal growth factor receptor-2.

**Table V.** Association among the expressions of VEGF, EGFR, p53, TOPO II and Ki67 in BCs [cases, (%)].

Indices	No.	EGFR		p53		TOPO II		Ki-67	
		(+)	P-value	(+)	P-value	(+)	P-value	(+)	P-value
VEGF									
(+)	154	132 (85.71)	0.19200	70 (45.45)	0.97900	82 (53.25)	0.73600	108 (70.13)	0.95500
(−)	132	102 (77.27)	-	64 (48.48)	-	74 (56.06)	-	92 (69.69)	-
EGFR									
(+)	234	-	-	110 (40.01)	0.93700	118 (50.43)	0.03600^a^	166 (70.94)	0.57600
(−)	52	-	-	24 (46.15)	-	38 (70.08)	4.40160	34 (65.38)	-
p53									
(+)	134	110 (82.09)	0.93700	-	-	92 (68.66)	0.00150^b^	112 (83.58)	0.00083^b^
(−)	152	124 (81.58)	-	-	-	64 (42.11)	10.12500	88 (57.89)	11.17360
TOPO II									
(+)	156	118 (75.64)	0.03600^a^	92 (58.97)	0.00150^b^	-	-	130 (83.33)	0.00013^b^
(−)	130	116 (89.23)	4.40160	42 (32.31)	10.12500	-	-	70 (53.85)	14.66030

a P≤0.05,

b P≤0.01, the χ^2^ values appeared when P≤0.05. BCs, breast cancers; VEGF, vascular endothelial growth factor; EGFR, epidermal growth factor receptor; TOPO II, type II topoisomerase.

**Table VI.** Association between the IHC approximate molecular subtypes of BC and clinicopathological characteristics [cases, (%)].

Clinicopathological characteristics	No.	Luminal A		Luminal B		HER2/neu subtype (non-Luminal)		Triple-negative	
		Yes	P-value	Yes	P-value	Yes	P-value	Yes	P-value
Age (years)									
≤50	140	28 (20.00)	0.7780	82 (58.57)	0.0020^b^	22 (15.71)	0.0040^b^	12 (8.57)	0.8320
>50	146	32 (21.92)	-	48 (32.87)	9.5156	54 (36.98)	8.2871	14 (9.59)	-
Tumor size (cm)									
≤2	164	38 (23.17)	0.4550	78 (47.56)	0.5580	34 (20.73)	0.0670	14 (8.54)	0.7890
>2	122	22 (18.03)	-	52 (42.62)	-	42 (34.43)	-	12 (9.84)	-
Lymph node metastasis									
Positive	134	22 (16.42)	0.2080	66 (49.25)	0.3920	40 (29.85)	0.4050	10 (7.46)	0.5250
Negative	152	38 (25.00)	-	64 (42.11)	-	36 (23.68)	-	16 (10.52)	-
Histological grade									
I-II	208	50 (24.04)	0.1423	100 (48.08)	0.3040	46 (22.12)	0.0490^a^	12 (5.77)	0.0240^a^
III	78	10 (12.82)	-	30 (38.46)	-	30 (38.46)	3.8841	14 (17.95)	5.0910

a P≤0.05,

b P≤0.01, the χ^2^ values appeared when P≤0.05. IHC, immunohistochemical; BC, breast cancer; HER2, human epidermal growth factor receptor-2.

**Table VII.** Definition and treatment strategies of BC subtypes according to the St. Gallen consensus in 2011.

Subtypes	Definition	Treatment strategies
Luminal A	ER+ and/or PR+, HER2 and Ki-67 low expression (<14%)	Endocrine therapy
Luminal B	Luminal B (HER2-negative)	Endocrine therapy ± cytotoxic therapy
	ER+ and/or PR+, HER2 and Ki-67 high expression (≥14%)	
	Luminal B (HER2/neu+)	
	ER+ and/or PR+, HER2 overexpression and Ki-67 uncertain	Endocrine therapy ± cytotoxic therapy + anti-HER2 therapy
Her2/neu	HER2/neu subtype (non-Luminal)	Cytotoxic therapy + anti-HER2 therapy
	ER and PR deficiency	
	HER2 overexpression or proliferation	
Triple-negative	ER and PR deficiency, HER2	Cytotoxic therapy

BC, breast cancer, ER, estrogen receptor; PR, progesterone receptor; HER2, human epidermal growth factor receptor-2.
